# Supplementary material for: High ferritin is associated with liver and bone marrow iron accumulation: Effects of 1-year deferoxamine treatment in hemodialysis-associated iron overload
Source: PLoS One. 2024 Aug 9;19(8):e0306255. doi: 10.1371/journal.pone.0306255 (PMC11315289; doi:10.1371/journal.pone.0306255)
Supplement: S1 File — (PDF) [file pone.0306255.s002.pdf]

Trial study protocol

RESEARCH STUDY PROTOCOL TITLE: "**High ferritin is associated with liver and bone marrow iron accumulation: effects of a 1-year deferoxamine treatment in hemodialysis-associated iron overload**"

Description of the procedures that will performed in the study protocol.

We will carry out a prospective 12-month study to evaluate by MRI, tissue Fe concentration in hepatic, cardiac, and bone in HD patients as well as the effect of the treatment with DFO in the same tissues after approval by the Ethics Committee and written informed consent. The inclusion criteria are ferritin levels >1,000 ng/ml, age > 18 years, on an HD schedule of 3 times/week for at least six months. The exclusion criteria are refusal to participate in the study, ethanol or drug abuse, claustrophobia, hepatic cirrhosis, active malignancy, HIV infection, hepatitis B and C, current use of steroids, presence of cardiac pacemakers or metallic cardiac valves, previous kidney transplant, and previous DFO treatment. The clinical and demographic parameters includ age, sex, race, primary cause of CKD, HD duration, and vascular access.

The magnetic resonance imaging (MRI) of the liver, heart, lumbar spine and iliac crest (right and left) will performed without contrast for the evaluation of iron overload in these locations based on the analysis of T2*, R2* and R2* Water and will acquired at the Instituto do Coração (InCor), Universidade de Sao Paulo with a 1.5 T MRI scanner (Achieva, Philips Healthcare, Amsterdam, Netherlands). The subjects will examined in the supine position. Cardiac and liver iron levels will measured using a 10–15 s breath-hold multiple-gradient echo T2* sequence. The following parameters will used for analyses: TR very short, flip angle 60°, 320 x 320 matrix, slice width 8 mm (gap 2 mm), 8 echoes gradient Time (TE) with minimum TE being 2.4 ms, and echo spacing 2.4 ms. The regions of interest (ROIs) will drawn using an axial section, with the largest liver area available, avoiding larger blood vessels and bile ducts. In the heart, the ROIs will drawn on the ventricular septum, excluding artifacts; in the iliac crests and lumbar spine L3 the ROIs will manually delimited by the radiologist, avoiding the cortical area. Processing of images in the liver, iliac crests, and lumbar spine L3 will

done by specific software (Dive-in - Magnepath-Perth, Australia) to quantify the R2*relaxation (R2*= 1000/T2* s-1) and afterward, an algorithm will used that calculate the relaxation value R2* for water (R2*Water)[1,2]. Calculation of liver iron concentration (LIC), liver normal parameters of T2* and R2*, and cardiac Fe overload will done according to previous studies [3,4,5]. We will analyzed the right the left iliac crest and lumbar spine of normal individuals and will determined the reference range (RR).

We will collected 10 to 20 ml of blood from a vein in the forearm of the fasting patients on the same day of the MRI for initial laboratory analysis and to evaluate response to DFO. Blood samples will centrifuged, aliquoted in cryovials, and stored at -80º. Serum levels of hemoglobin (Hb) (reference range (RR)=13.5-17.5 g/dL), Fe (RR=65-175 µg/dL), transferrin saturation (RR=20-40%), ferritin (RR=13-150 ng/mL), total calcium (tCa) (RR=8.5-10.5 mg/dL), ionized calcium (iCa) (RR=4.6–5.3 mg/dL), phosphate (RR=2.3-4.7 mg/dL), total alkaline phosphatase (AP) (RR=35–104 U/L for females and 40–129 U/L for males), and C-reactive protein (CRP) (RR<0.5 mg/dL-immunoturbidimetric method), were analyzed using standard laboratory techniques. Intact PTH (Immulite; DPC-Biermann, Bad Nauheim, Germany, RR=10–65 pg/mL), 25(OH) vitamin D (immunoassay RR=30-100 ng/ml), intact FGF 23 (ELISA FGF 23 Human intact-Quidel San Diego, CA, USA, RR=18-73 pg/ml), C-terminal FGF-23 (Human FGF-23 C-Term, ELISA, Quidel San Diego, CA, USA, RR=21.6-91.0 RU/L), and Human Hepcidin (Quantikine ELISA, kit R&D Systems Inc, Minneapolis, MN, USA, RR=not determined) will used according to the manufacturers' instructions.

We also will performed a transiliac bone biopsy one day after the Magnetic Resonance Imaging and blood collection, using a 7-mm Bordier trephine after a course of double-labelling tetracycline (20 mg/kg/day) for 3 days, separated by an interval of 10 days. The biopsy will performed 2–5 days after the last dose of antibiotics. The specimens will fixed in 70% ethanol, dehydrated, and embedded in methyl methacrylate. Undecalcified 5-µm-thick sections will cut using a Polycut S microtome equipped with a tungsten carbide knife (Leica, Heidelberg, Germany). Some sections will stained with 0.1% toluidine blue, pH 6.4, and unstained 10-µm slices will obtained for analysis of dynamic parameters under a microscope with ultraviolet light. Solochrome azurine and Perls Prussian blue staining will used to evaluate aluminum and Fe deposits, respectively. The number of iron-stained cells per square millimeter of

bone marrow will systematically counted in the complete bone marrow area. The parameters will measured and nomenclature described were: total number of cells iron positive (N.Cells Fe+, nº); total number of cells iron positive per square millimeter of marrow area (Cells Fe+/Ma.Ar, nº/mm2). Histomorphometric analyses will performed using a semiautomatic image analyzer and Osteomeasure software (Osteometrics, Inc., Atlanta, GA, USA). The static and dynamic parameters will reported using the nomenclature recommended by the American Society for Bone and Mineral Research [6]. We will measured: bone volume (BV/TV, %); trabecular thickness (Tb.Th, µm); trabecular separation (Tb.Sp, µm); trabecular number (Tb.N); fibrosis volume (Fb.V, %) osteoid volume (OV/BV, %); osteoid thickness (O.Th, µm); osteoid surface (OS/BS, %); osteoblast surface (Ob.S/BS, %); eroded surface (ES/BS, %); osteoclast surface (Oc.S/BS, %); mineral apposition rate (MAR, µm/day); mineralizing surface (MS/BS, %); bone formation rate (BFR/ BS, µm3 /µm2 /day) and mineralization lag time (Mlt, days), cortical thickness (CT.Th, µm) and cortical porosity (Ct.Po, %). The reference ranges will used for static and dynamic parameters will obtained from our normal laboratory controls [7] and according to Melsen [8], respectively. The bone histology will categorized according to the proposed Turnover/Mineralization/Volume (TMV) classification [9].

After the end of the blood and imaging tests, infusion of desferoxamine (DFO) will started 5mg/kg diluted in saline 0,9% 100ml, with a weekly dose, performed during the last hour of renal replacement therapy, in the second hemodialysis session of the week at the beginning of the study and for a period of one year

In the end of 12 months of DFO use, the patients who will remained in the study underwent will MRI again from the same sites performed at the beginning of the study, blood collection on the same day of MRI, and transiliac bone biopsy one day after MRI and blood collection for analysis and description of the results.

Data will reported as number (frequency), mean ± SD or median (25-75) as appropriate. Differences between group characteristics will tested with the Chi-square test for categorical variables and the Fisher's exact test for continuous variables. The prospective analysis will limited to patients that completed the 12-month follow-up, using the Chi-square test for categorical variables and Fisher's exact test for continuous variables, as appropriate. The Spearman correlation coefficient will assessed relationships between independent variables. All statistical tests will two-tailed with a

significance level of 5%. Analyses will performed with GraphPad Prism 8 and SPSS for Windows 21.0 program.

References

1. Henninger B. Demystifying liver iron concentration measurements with MRI. *EurRadiol* 2018; 28: 2535-2536.

2. Kuhn JP, Hernando D, Munoz del Rio A, *et al.* Effect of multipeak spectral modeling of fat for liver iron and fat quantification: correlation of biopsy with MRimaging results. *Radiology* 2012; 265: 133-142.

3. Wood JC, Enriquez C, Ghugre N, *et al.* MRI R2 and R2* mapping accurately estimates hepatic iron concentration in transfusion-dependent thalassemia andsickle cell disease patients. *Blood* 2005; 106: 1460-1465.

4. Garbowski MW, Carpenter JP, Smith G, *et al.* Biopsy-based calibration of T2* magnetic resonance for estimation of liver iron concentration and comparison withR2 Ferriscan. *J Cardiovasc Magn Reson* 2014; 16: 40.

5. Carpenter JP, He T, Kirk P, *et al.* On T2* magnetic resonance and cardiac iron. *Circulation* 2011; 123: 1519-1528.

6. Dempster DW, Compston JE, Drezner MK, *et al.* Standardized nomenclature, symbols, and units for bone histomorphometry: a 2012 update of the report of theASBMR Histomorphometry Nomenclature Committee. *J Bone Miner Res* 2013; 28: 2-17.

7. Dos Reis LM, Batalha JR, Munoz DR, *et al.* Brazilian normal static bone histomorphometry: effects of age, sex, and race. *J Bone Miner Metab* 2007; 25:400-406.

8. Melsen F, Mosekilde L. Trabecular bone mineralization lag time determined by tetracycline double-labeling in normal and certain pathological conditions. *ActaPathol Microbiol Scand A* 1980; 88: 83-88.

9. Moe S, Drueke T, Cunningham J*, et al.* Definition, evaluation, and classification ofrenal osteodystrophy: a position statement from Kidney Disease: Improving Global Outcomes (KDIGO). *Kidney Int* 2006; 69: 1945-1953.
