# Supplementary material for: High ferritin is associated with liver and bone marrow iron accumulation: Effects of 1-year deferoxamine treatment in hemodialysis-associated iron overload
Source: PLoS One. 2024 Aug 9;19(8):e0306255. doi: 10.1371/journal.pone.0306255 (PMC11315289; doi:10.1371/journal.pone.0306255)
Supplement: S2 File — (PDF) [file pone.0306255.s003.pdf]

S2 Methods

Bone biopsy and histomorphometry

Patients were submitted to a transiliac bone biopsy using a 7-mm Bordier trephine after a course of double-labelling tetracycline (20 mg/kg/day) for 3 days, separated by an interval of 10 days. The biopsy was performed 2–5 days after the last dose of antibiotics. The specimens were fixed in 70% ethanol, dehydrated, and embedded in methyl methacrylate. Undecalcified 5-μm-thick sections were cut using a Polycut S microtome equipped with a tungsten carbide knife (Leica, Heidelberg, Germany). Some sections were stained with 0.1% toluidine blue, pH 6.4, and unstained 10-μm slices were obtained for analysis of dynamic parameters under a microscope with ultraviolet light. Solochrome azurine and Perls Prussian blue staining were used to evaluate aluminum and Fe deposits, respectively. The number of iron-stained cells per square millimeter of bone marrow was systematically counted in the complete bone marrow area. The parameters measured and nomenclature described were: total number of cells iron positive (N.Cells Fe^{+}, n^{o}); total number of cells iron positive per square millimeter of marrow area (Cells Fe^{+}/Ma.Ar, n^{o}/mm^{2}).

Histomorphometric analyses were performed using a semiautomatic image analyzer and Osteomeasure software (Osteometrics, Inc., Atlanta, GA, USA). The static and dynamic parameters were reported using the nomenclature recommended by the American Society for Bone and Mineral Research[1]. We measured: bone volume (BV/TV, %); trabecular thickness (Tb.Th, μm); trabecular separation (Tb.Sp, μm); trabecular number (Tb.N); fibrosis volume (Fb.V, %) osteoid volume (OV/BV, %); osteoid thickness (O.Th, μm); osteoid surface (OS/BS, %); osteoblast surface (Ob.S/BS, %); eroded surface (ES/BS, %); osteoclast surface (Oc.S/BS, %); mineral apposition rate (MAR, μm/day); mineralizing surface (MS/BS, %); bone formation rate (BFR/ BS, μm^{3} /μm^{2} /day) and mineralization lag time (Mlt, days), cortical thickness (CT.Th, μm) and cortical porosity (Ct.Po, %). The reference ranges used for static and dynamic parameters were obtained from our normal laboratory controls[2] and according to Melsen[3], respectively. The bone histology was categorized according to the proposed Turnover/Mineralization/Volume (TMV) classification[4].

Magnetic Resonance Imaging

MRI of the liver, heart, iliac crests, and lumbar spine were acquired at the Instituto do Coração (InCor), Universidade de Sao Paulo with a 1.5 T MRI scanner (Achieva, Philips Healthcare, Amsterdam, Netherlands). The subjects were examined in the supine position. Cardiac and liver iron levels were measured using a 10–15 s breath-hold multiple-gradient echo T2* sequence. The following parameters were used for analyses: TR very short, flip angle 60°, 320 x 320 matrix, slice width 8 mm (gap 2 mm), 8 echoes gradient Time (TE) with minimum TE being 2.4 ms, and echo spacing 2.4 ms.

The regions of interest (ROIs) were drawn using an axial section, with the largest liver area available, avoiding larger blood vessels and bile ducts. In the heart, the ROIs drawn on the ventricular septum, excluding artifacts; in the iliac crests and lumbar spine L3 the ROIs were manually delimited by the radiologist, avoiding the cortical area.

Processing of images in the liver, iliac crests, and lumbar spine L3 was done by specific software (Dive-in - Magnepath-Perth, Australia) to quantify the R2*relaxation (R2*= 1000/T2* s-1) and afterward, an algorithm was used that calculate the relaxation value R2* for water (R2*Water)[5,6]. Calculation of liver iron concentration (LIC), liver normal parameters of T2* and R2*, and cardiac Fe overload was done according to previous Studies[7,8,9].

We analyzed the right the left iliac crest and lumbar spine of 6 normal individuals (4 men and 2 women with a mean age of 30 years) and determined the reference range (RR). Iliac crest right R2*Water was 108.39 ± 29.13, R2* 84.95 ± 13.45. Iliac crest left R2*Water was 108.45 ± 26.0 and R2* was 85.91 ± 12.6. Lumbar spine L3 R2*Water 102.18 ± 20.8, and R2* 87.95 ± 12.8. We considered up to 1 SD from the average as normal, as described in S3.

Statistical analysis

Data are reported as number (frequency), mean ± SD or median (25-75) as appropriate.

Differences between group characteristics were tested with the Chi-square test for categorical variables and the Fisher's exact test for continuous variables. The prospective analysis was limited to patients that completed the 12-month follow-up, using the Chi-square test for categorical variables and Fisher's exact test for continuous variables, as appropriate. The Spearman correlation coefficient assessed relationships between independent variables. All statistical tests were two-tailed with a significance level of 5%. Analyses were performed with GraphPad Prism 8 and SPSS for Windows 21.0 program.

References

1. Dempster DW, Compston JE, Drezner MK, *et al.* Standardized nomenclature, symbols, and units for bone histomorphometry: a 2012 update of the report of the ASBMR Histomorphometry Nomenclature Committee. *J Bone Miner Res* 2013; **28:** 2-17.
2. Dos Reis LM, Batalha JR, Munoz DR, *et al.* Brazilian normal static bone histomorphometry: effects of age, sex, and race. *J Bone Miner Metab* 2007; **25:** 400-406.
3. Melsen F, Mosekilde L. Trabecular bone mineralization lag time determined by tetracycline double-labeling in normal and certain pathological conditions. *Acta Pathol Microbiol Scand A* 1980; **88:** 83-88.
4. Moe S, Drueke T, Cunningham J, *et al.* Definition, evaluation, and classification of renal osteodystrophy: a position statement from Kidney Disease: Improving Global Outcomes (KDIGO). *Kidney Int* 2006; **69:** 1945-1953.
5. Henninger B. Demystifying liver iron concentration measurements with MRI. *Eur Radiol* 2018; **28:** 2535-2536.
6. Kuhn JP, Hernando D, Munoz del Rio A, *et al.* Effect of multipeak spectral modeling of fat for liver iron and fat quantification: correlation of biopsy with MR imaging results. *Radiology* 2012; **265:** 133-142.
7. Wood JC, Enriquez C, Ghugre N, *et al.* MRI R2 and R2* mapping accurately estimates hepatic iron concentration in transfusion-dependent thalassemia and sickle cell disease patients. *Blood* 2005; **106:** 1460-1465.
8. Garbowski MW, Carpenter JP, Smith G, *et al.* Biopsy-based calibration of T2* magnetic resonance for estimation of liver iron concentration and comparison with R2 Ferriscan. *J Cardiovasc Magn Reson* 2014; **16:** 40.
9. Carpenter JP, He T, Kirk P, *et al.* On T2* magnetic resonance and cardiac iron. *Circulation* 2011; **123:** 1519-1528.
