# Supplementary material for: High ferritin is associated with liver and bone marrow iron accumulation: Effects of 1-year deferoxamine treatment in hemodialysis-associated iron overload
Source: PLoS One. 2024 Aug 9;19(8):e0306255. doi: 10.1371/journal.pone.0306255 (PMC11315289; doi:10.1371/journal.pone.0306255)
Supplement: S1 Table — (PDF) [file pone.0306255.s004.pdf]

Supplemental Tables

S1 Table. Characteristics of patients.

Parameter	N = 28	Minimal - Maximal
Age, years	56 ± 13	30 to 81
Male gender, n (%)	17 (60.7)	-
Non-white, n (%)	16 (57.1)	-
Etiology of CKD, n (%)		
Nephrosclerosis	8 (28.6)	
Diabetes	13 (46.4)	
Other	7 (25.0)	
Vascular access, n (%)		
Arteriovenous fistulae	22 (78.6)	
Catheter	6 (21.4)	
Weight, kg	70.5 ± 12.9	50.6 to 97.7
Body Mass Index, kg/m^2	26.5 ± 5.1	19.9 to 42.2
Dialysis vintage, months	36 (25, 54)	9 to 108
Hemoglobin, g/dl	11.5 ± 2.0	8.7 to 15.5
Fe, µg/dl	111.5 ± 49.0	46 to 227
Transferrin saturation, %	42 (32-61)	22 to 93
Serum ferritin, ng/ml	1,612 ± 599	1,039 to 3,172
Hepcidin, ng/ml	192 ± 45	87 to 343
C-reactive protein, mg/dl	4.9 (2.6-11.9)	0.3 to 23.8
Ionized Calcium, mg/dl	4.95 ± 0.29	4.35 to 5.59
Total Calcium, mg/dl	9.6 ± 0.6	8.1 to 10.8
Phosphate, mg/dl	5.2 ± 1.6	1.6 to 8.9
PTH, pg/ml	283 (140-681)	51 to 3,926
25-(OH) vitamin D (ng/ml)	25.2 ± 11.4	7.1 to 57.0
cFGF-23, RU/ml	1,231 (672-6,378)	242 to 13,665
iFGF-23, pg/ml	1,292 (440-5,186)	15 to 10,387
Alkaline phosphatase, U/L	129 (99-181)	62 to 1,050
**Medications in use**		
Erythropoietin, UI/kg/week	160 (84-190)	0 to 404
IV iron, mg/month	258 (200-508)	92 to 1,333
Calcitriol (µg/week)	0 (0-2.75)	0 to 6
Cinacalcet (mg/day)	0 (0-0)	0 to 90
Sevelamer (g/day)	4.8 (4.8-7.2)	0 to 7.2

Values are expressed as the mean and standard deviation or median (25,75); CKD, chronic kidney disease; Fe iron; PTH, parathyroid hormone; FGF23c, carboxy-terminal fibroblast growth factor; FGF23i, intact fibroblast growth factor 23; IV, intravenuous.
