# Supplementary material for: High ferritin is associated with liver and bone marrow iron accumulation: Effects of 1-year deferoxamine treatment in hemodialysis-associated iron overload
Source: PLoS One. 2024 Aug 9;19(8):e0306255. doi: 10.1371/journal.pone.0306255 (PMC11315289; doi:10.1371/journal.pone.0306255)
Supplement: S2 Table — (PDF) [file pone.0306255.s005.pdf]

S2 Table. Bone histomorphometric parameters.

Parameter	N = 28	Minimal - Maximal
BV/TV (%)	19.16 ± 6.99	9.7 to 41.1
Tb.Th (µm)	117.4 ± 24.62	77.7 to 184
Tb.Sp (µm)	510.3 ± 172.4	101 to 781
Tb.N (/mm)	1.64 ± 0.56	0.85 to 3.71
Fb.V/TV (%)	0.085 (0-0.42)	0 to 17.4
OV/BV (%)	6.3 (1.4-8.9)	0.23 to 30.4
O.Th (µm)	9.6 ± 4.0	4.3 to 18.4
OS/BS (%)	38.37 ± 23.9	6.7 to 86.1
Ob.S/BS (%)	11.8 (4.3-19.2)	1.4 to 56.3
ES/BS (%)	7.38 ± 4.2	2.1 to 17.7
Oc.S/BS (%)	0.98 (0.4- 2.0)	0.07 to 4.27
MAR (µm/d)	0.90 ± 0.37	0.22 to 1.58
MS/BS (%)	7.46 ± 5.17	0.57 to 19.69
BFR/BS ($\mu m^3/\mu m^2/d$)	0.06 (0.015-0.11)	0 to 0.25
Mlt (d)	61 (38-197)	5 to 464
CT.Th (µm)	626 (527-850)	352 to 1,437
Ct.Po (%)	10.3 (6.9-15.7)	2.8 to 31.4
N.Ot (nº)	719 (418-1,113)	80 to 3,447
N.Cells Fe + (nº)	5554 ± 3404	309 to 13,955
Ma.Ar (mm^2)	28.7 ± 10.5	10.8 to 57.5
Cells Fe+/Ma.Ar (n°/mm^2)	190.4 (120.7-293.8)	14.68 to 457.8

Values are expressed as the mean and standard deviation or median (25/75);
