# Supplementary material for: High ferritin is associated with liver and bone marrow iron accumulation: Effects of 1-year deferoxamine treatment in hemodialysis-associated iron overload
Source: PLoS One. 2024 Aug 9;19(8):e0306255. doi: 10.1371/journal.pone.0306255 (PMC11315289; doi:10.1371/journal.pone.0306255)
Supplement: S3 Table — (PDF) [file pone.0306255.s006.pdf]

S3 Table. Magnetic Resonance Imaging.

Parameter	N = 28	Minimal - Maximal	Reference range
Cardiac T2*, ms	48.7 ± 26.1	27.9 to 147	> 20 ms
Liver LIC mg/g	4.4 ± 2.3	1.5 to 10.4	< 2.0 mg/g
Liver T2*, ms	6.9 ± 3.0	2.6 to 14.5	> 15.4 ms
Liver R2* Water, Hz	162 ± 69	67 to 325	
Liver R2*, Hz	174 ± 81	69 to 386	< 65 Hz
Lumbar spine R2* Water, Hz	168 ± 44	51 to 241	< 128 Hz
Lumbar spine R2*, Hz	110 ± 35	15 to 176	< 87 Hz
Right iliac crest R2* Water, Hz	161 ± 47	94 to 304	< 139
Right iliac crest R2*, Hz	116 ± 25	66 to 160	< 107
Left iliac crest R2*, Water, Hz	160 ± 48	96 to 277	< 139
Left iliac crest R2*, Hz	115 ± 28	60 to 168	< 107

Values are expressed as the mean and standard deviation;
