# Supplementary material for: High ferritin is associated with liver and bone marrow iron accumulation: Effects of 1-year deferoxamine treatment in hemodialysis-associated iron overload
Source: PLoS One. 2024 Aug 9;19(8):e0306255. doi: 10.1371/journal.pone.0306255 (PMC11315289; doi:10.1371/journal.pone.0306255)
Supplement: Attachment — Submitted filename: Response to Reviewers.docx [file pone.0306255.s008.docx]

RE: PONE-D-24-04153R1

Manuscript entitled **"High ferritin is associated with liver and bone marrow iron accumulation: effects of 1- year deferoxamine treatment in hemodialysis associated iron overload”**

Thank you for encouraging us to submit a revised version of the above-titled paper for re-evaluation by your expert Reviewers.  We thank the reviewers for their insightful comments, which have been addressed in a manuscript that we believe is now improved.

Enclosed, please find our responses to reviewers highlighting the changes made to our manuscript conforming to the style of your journal. If the Editors would like further clarification, we would be happy to provide it wherever specified.

Sincerely,

Vanda Jorgetti

Melani Custodio

**Response to Academic Editor**

** 1.* Please review your reference list to ensure that it is complete and correct.

*R: Thank you for your observation. We have review and included the correct references in line 148 and 202.*

**Responses to the Reviewer 1**

* This revised version has considered reviewers’ comments and advice ad seems suitable for publication in PLOS ONE. One minor remark: authors please explain the acronym TMV.

*R.: Thank you for your observation. We have explained the acronym TMV in line 185-187.*

**Responses to the Reviewer 2**

* Thank you for your previous responses. I would request one chance to Table 4 - this is presented as if the data are not paired - please instead cross tabulate the values at the different timepoints- the data currently presented is therefore in the marginal data, but one can see the paired data allowing clarity of testing.

*R: Thank you for your suggestion. We agree and have included this information in Figure 5*
